# Determining skill mix and optimal multidisciplinary team composition: A scoping review

**DOI:** 10.1177/08404704241293095

**Published:** 2024-11-05

**Authors:** Donna Meadows, Joanne Maclaren, Alec Morton, Darcy Ross

**Affiliations:** 1 University of Strathclyde, Glasgow, Scotland, United Kingdom.; 2 Island Health, Victoria, British Columbia, Canada.; 3 Island Health, Nanaimo, British Columbia, Canada.

## Abstract

Holistic care models aligned to population care needs are needed to help leaders shed pre-existing mindsets when determining skill mix and Multidisciplinary Team (MDT) composition. Using a PRISMA flowchart, a narrow eligibility criterion, and a research question, this scoping review resulted in 9 frameworks/models published between January 2000 and September 2023. Analysis showed common methodological elements such as a population needs-based approach, a systematic process, engagement, three or more professions reporting task or competency level analysis, change advocacy, and reliance on population and workforce supply data. Key system enablers were sponsorship, access to population needs-based and workforce supply data, a learning management system for MDT development, and health human resource policies and governance to drive health system redesign to distribute an equitable workforce. This scoping review offers health leaders and policy-makers options and next-step considerations to inspire fresh thinking for making evidence-informed decisions about skill mix and MDT composition.

## Introduction

Despite many studies and systematic literature reviews that highlight today’s complex challenges for leaders, there is an unexplored number of practical Multidisciplinary Team (MDT) frameworks published in the last two decades that inform decision-making in determining skill mix, optimal design, and team composition. Although delivering high quality, efficient care for increasingly complex and diverse populations needs a broader range of care activities and roles performed as part of a team, many systematic reviews on skill mix focus on single professions or on a narrow set of outcomes^1^ making it difficult to find studies that include three or more professions or roles. The objectives of this scoping review are to map and summarize the published MDT frameworks or models that analyze skill mix from that broader perspective, consider three or more professions,^1-5^ and can report on roles and explicit tasks performed.^2^ An analysis of the frameworks/models includes key findings, learnings, and next-step considerations.

## Purpose and why now?

Global experience suggests healthcare delivery models of care are not working and need to be reshaped or redesigned with new thinking.^6-11^ When designing or re-designing MDTs, health leaders need evidence-informed frameworks or models that are flexible with varying populations to guide skill mix and team composition decisions. Without a deep understanding of the care needed, and the tasks and skill mix required to meet those needs, leaders may tend to repeat ingrained decision-making and narrowly defined hiring processes. Expanded services, limited workforce availability, demographic shifts, and more complex healthcare needs are driving the need to rethink earlier workforce decisions and strategies that have been successful in the past but no longer meet population needs. As current models of care break down, leaders are looking for more holistic, population-focused frameworks or models of care as alternatives to their mental maps.

## Method

Electronic databases searched were CINAHL, PubMed, MEDLINE, and Google Scholar. Records were filtered between January 2000 and September 2023, included all countries and available in English. Search terms included combined Medical Subject Headings (MeSH) with free text words including: “determining skill mix in healthcare teams.”

A scoping review approach enabled drawing on a broader range of literature but did not require assessing the quality of the studies, which would be challenging given the lack of authoritative quality criteria for articles of this type. The review also offered a dual-search strategy to split the records published pre- and post-COVID-19 pandemic with the purpose of finding newly developed MDT frameworks informed by pandemic knowledge. The first search included reviews, systematic and Cochrane reviews, and Randomized Clinical Trials (RCTs) published pre-pandemic (January 2000-December 2019). The latter search (January 2020-September 2023) included unpublished grey literature, policy briefs, textbooks, editorials, comparative and observational studies, and some non-peer reviewed studies intended to capture a broader scope of potential frameworks derived from early published pandemic learnings. Two reviewers performed full-text screening and data charting.

After screening titles and abstracts, snowballing was used to find other relevant studies. At the eligibility stage, full text was reviewed and considered if: the record revealed a framework, model, tool, method, or innovative approach to address care needs across a population/care setting; described three or more professions or types of healthcare roles; and answered the research question: What are the frameworks or models published in the health sciences research literature that can be used to inform and determine skill mix and multidisciplinary team-based care composition?

The inclusion and exclusion criteria used are described in Table 1.

**Table 1.** Inclusion and exclusion criteria.

PRISMA	Inclusion criteria	Exclusion criteria
Identification	• Included records between Jan. 2000 and Sept. 2023	• Did not have “determine skill mix” in its search terms
	• If the record was from 2000 to 2019, it had to be a review, a systematic review, a Cochrane review, or a RCT from PUBMED	
	• If the record was from Jan. 2020 to Sept. 2023, it could be any type of published research article including grey literature	
	• English language and any country	
	• Any area of healthcare including dentistry	
	• Has “determine skill mix” in its search terms	
Screening	• Title and abstract described a framework or model to determine skill mix	• Title and abstract did not allude to include a model or framework
Eligibility	• Full-text review revealed a framework, model, tool, method, or innovative approach for determining skill mix to address care needs across a population/care setting	• Full-text review focused on only one or two professions or types of healthcare roles
	• Included three or more professions or healthcare roles	• Did not answer research question
	• Explicitly reported skill mix analysis of tasks required to address care needs	
	• Article/Study could answer research question	
Included	• Article/Study met inclusion and could contribute to answering research question	

## Results

**Figure 1.**
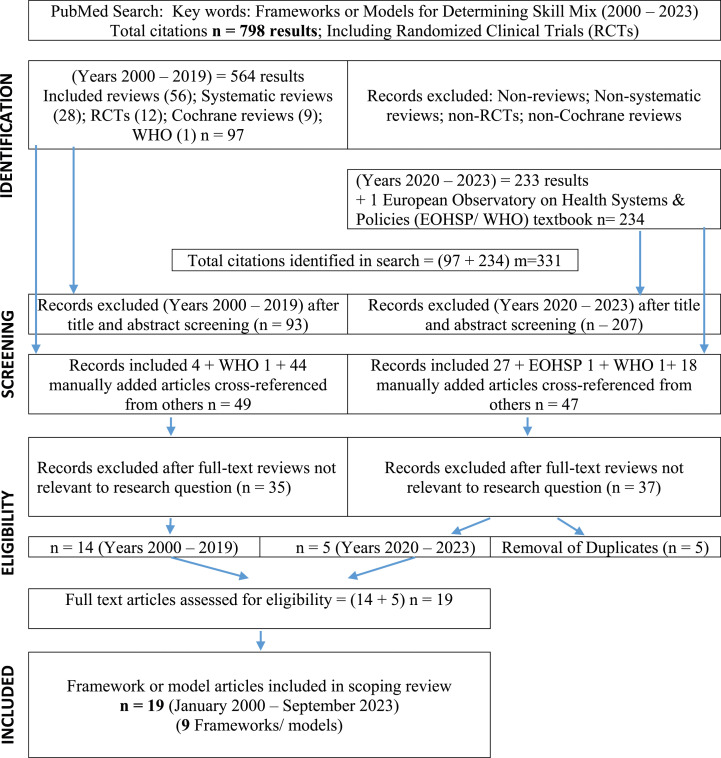
PRISMA literature flow chart.

The initial search found 798 records between January 2000 and September 2023. Upon screening titles and abstracts, and after additional articles were cross-referenced and manually added, 96 eligible records were found between the two sub-searches. Full-text versions were assessed using the inclusion-exclusion criteria and the research question. After removal of duplicates, 77 records were excluded leaving 19 eligible articles. Within these, this scoping review revealed 9 frameworks or models that were all developed pre-COVID-19 pandemic (before 2020), even though some were published after the pandemic (see Figure 1: PRISMA literature flow chart).

## Key findings

Since all frameworks that met this review’s criteria were developed before 2020, this may mean it is still early to see newly developed MDT frameworks that incorporated pandemic learnings in published literature.

The 9 frameworks or models’ drivers ranged from a need to better understand changing population needs and match the right skills and competencies to inform workforce role development and education, address Health Human Resources (HHR) workforce planning and emerging needs to support equitable distribution of workload and productivity in care delivery, and to support leaders and developing care teams to address service gaps and improve patient care/experience.

Table 2 provides a summary of the 9 model of care frameworks within the 19 reference articles that met the criteria.

**Table 2.** Model of care framework summary.

Framework/Year/Study/Author/Country	Additional published evidence of MDT Model	Purpose	Professions/Population/Care setting	Steps
Integrated Needs-Based Framework, 1993 Birch et al., 2007^5^ Canada	Pandemic planning Murphy et al., 2017^12^; Birch et al., 2021^13^	Population needs-based framework/model for healthcare service and HHR planning including potential future influenza pandemic	All professions Oral health and pandemic planning All care settings	1. Identify population (who are we caring for?) 2. Epidemiology (expected levels of risk of that population) 3. Evidence-based services (what services do we plan to provide for different risk/health groups?) 4. Productivity (how do we plan to provide those services?
The Workload Indicators of Staffing Need Tool (WISN)/1998, 2010 (revision) World Health Organization, 2023^14^ Multiple countries	Wundavali et al, 2019^15^; Okoroafor et al., 2019^16^	Well managed health workforce with equitable distribution of workload and better productivity for countries to meet their health commitments A manual for two groups: directors and senior managers to authorize and supervise application and managers and health professionals implement/provide input	Nursing and support staff All populations All care settings including emergency dept., primary care, and pandemic planning	1. Determine cadre (profession) and health facility 2. Estimate available working time 3. Determine workload components 4. Define activity standards 5. Establish standard workloads 6. Calculate allowance factors 7. Determine staff requirement based on WISN 8. Analyze and interpret results
Calderdale Framework, 2008 Smith and Duffy, 2010/2013^17^ Australia	Pighills et al., 2015^18^; Patterson et al., 2015^19^; Kaltner et al., 2017^20^ Multiple countries including Calderdale NHS Trust, UK; Australia; New Zealand	Workforce framework/model for skill sharing across professions and competency training for a non-regulated workforce‐delegating tasks and core competency set for nursing support staff working in Mental Health and Substance Use (MHSU) programs. Skill sharing ends up like uni-professional	Non-regulated, nursing support staff, and allied health All populations including cancer care (rural/remote) All care settings including rehabilitation services	1. Awareness raising 2. Service analysis 3. Task analysis (focus on risk management) 4. Competency identification (focus on quality) 5. Supporting systems (focus on governance) 6. Training (focus on staff development) 7. Sustaining (focus on embedding and monitoring)
3-Stage Computer Model/Linear Programming/Demand and Supply Model, 2010 Demand Model, Monte Carlo Simulation Model Gallagher et al., 2010^21^ UK	Demand Model, UK: Gallagher et al., 2013^22^	Model to inform role development and education in dentistry workforce as population ages, retains their teeth and care becomes more complex, and preventative care	All dental team professionals All populations Dentistry care settings	1. Analyze demand 2. Project workforce supply 3. Use operational research methods to model skill mix
Workforce Evidence-Based (WEB) Model 2011 Segal and Leach, 2011^23^ Australia	South Australia study (*The Lancet*) Segal et al., 2018^24^	Workforce planning model to estimate the health workforce team (i.e., skill mix and hours) with varying populations and emerging needs to support delivery of best-practice interdisciplinary care	All professions All populations including tertiary mental health needs All care settings	1. Competency and skill-based needs assessment 2. Regional workforce based on care requirements 3. Cost clinical workforce 4. Service delivery and policy implications
Six Steps Methodology to Integrated Workforce Planning, 2015 Skills for Health, 2015c^25^ United Kingdom (UK)		Workforce planning methodology to understand desirable workforce with right competencies and skills for patient populations	All professions All populations All care settings	1. Define the plan 2. Map service change 3. Define required workforce 4. Understand workforce availability 5. Plan to deliver required workforce 6. Implement, monitor, refresh
The Care Process Framework, 2015 Meadows et al., 2023 ^26^ Canada		To provide leaders with novel and practical approach to care model development‐to match population care needs to skills mix required	All professions All populations All care settings	1. Establish need 2. Initiate a plan 3. Analyze current state—Rapid Task Analysis (RTA) 4. Map desired future state (RTA and audience analysis—match competency to tasks) 5. Develop recommendations and implementation plan (consider policy implications) 6. Implement change 7. Consolidate and evaluate change(s) 8. Sustain and monitor for Continuous Quality Improvement (CQI)
The Total Resident Aged and Restorative Staffing and Skills Mix Matrix Model©, 2016 Willis et al., 2016^27^ Australia		Understand skills and competencies to meet population needs and then create new carer role mixes, pathways, and options to best achieve team composition	Nursing and support staff Seniors care Long-term care setting	1. Calculate direct nursing and personal care time per intervention per resident x frequency per shift 2. Calculate indirect nursing and personal care time per intervention per resident x frequency per shift 3. Determine role mix, pathways, and options to deliver care
Primary and Community Care (PACC) mapping, 2019 Price et al., 2023^29^ Price et al., 2020^28^ Canada		To describe team mapping method as a tool to support developing primary care teams to address service gaps and help communities develop local solutions to improve patient care/experience	All professions Primary care population Primary care setting but potential to use in other care settings	1. Circle of care modelling 2. Business origami 3. Design charrettes

This review revealed the following common methodological elements across the framework/models: start with a population needs-based analysis; include a standardized, step-by-step systemic process; engage patient partners and subject matter experts/care team to better understand the care needed; map care needs at the task/competency level analysis of care; incorporate or acknowledge a change element process; and rely on population and workforce supply data.

Collaboration, gaining sponsorship, and engagement with leaders and clinical team members were key elements in the frameworks.^5,12-29^ Most leaned on clinical staff and partners to inform care pathways, competencies, and learning gaps, and to provide information to complete their calculations. Quality care outcomes, along with patient and staff satisfaction, improved when clinical staff were engaged in designing and re-designing MDTs, including workforce planning frameworks, reinforcing the necessity of engaging with staff. Some frameworks offer facilitator packages and other training materials available on their web sites or through a consultative process.^5,14,17,21,25-29^ One framework highlighted reduced trust when too narrow a representation of clinicians, community partners (such as First Nations), and patient partners participated in the process.^28,29^

Using a standardized practice change guide^26^ helped build team readiness for change and maximize success. Changing management resources to redesign MDTs improved success and resulted in new ways of working.^5,14,17,25,26^

Analysis also illuminated essential system enablers that enhanced practical and effective operational implementation of the frameworks by health leaders. These have implications for practice, policy, and research.

### Essential system enablers

• Promote strong sponsorship at all levels; macro‐ (healthcare policy), meso‐ (healthcare organization/institute), and micro‐ (patient care) levels of the health system.^5,12,13,17,25-27^
• Provide ready access to current and longitudinal population health and needs-based data.
• Incorporate detailed human resource workforce supply data that includes full-time equivalent skills and competencies.
• Maintain a learning management system to support MDT competency development and maintenance, performance support tools and resources.
• Align policies and governance that drive health system redesign to enable MDT learning, flexibility, and adaptability across the system.

## Discussion

With the HHR challenges and current complexities of care, many leaders find it difficult to address service needs with today’s policies and models. This scoping review provides a summary and analysis of the evidence-informed MDT frameworks published in the literature in the last two decades that are effective, relevant, and available now for study and use. The Canadian Quality & Patient Safety Framework for Health Services Area Action 3.2 that provides expectations for health leaders’ states: “Human resources are effectively matched to population needs where health leaders are to determine a needs-based human resource allocation strategy, including an appropriate skill mix for the workforce.”^30^

Across health and dental sectors involving framework authors, there was agreement that more holistic person-centred approaches to care by multiple professions and skill mixes are needed. For example, Winkelmann et al. emphasized the importance of shifting away from “fragmented, disease-based models of care” to integrated, holistic approaches.^6^ Similarly, Brocklehurst and Macey recognized that as dentistry shifts from a “cure to a care culture,” the redesigned model would include more diverse and varied roles.^7^

Using an MDT framework to determine population care needs mapped to goals of care, levels of health complexity and varying skill mix, could address distribution gaps such as geographical or recruitment challenges in certain areas of practice. For instance, the World Health Organization (WHO) mentions workforce distribution is poorly balanced between rural and urban populations and across primary, secondary, and tertiary levels of care.^14^ Using a MDT framework could also enable local policy level directives such as telemedicine, virtual services, or different provider payment/funding approaches.^30^

All framework authors recognized changing health demographics and related care needs and/or HHR challenges as drivers for their research, while some highlighted past hirings as a barrier to shift who gets hired for which roles and letting go of past assumptions about MDTs. Some traditional HHR planning methods resulted in unmet needs because of little consideration for population health needs,^7,12-14,24,25,27^ due to a focus usually on only one type of profession,^7^ and/or unmatched service needs to HHR requirements.^14^ The common elements of the 9 frameworks suggest flexibility with varying populations to guide MDT composition decisions.

Our experience and reflections doing this scoping review revealed three key insights relevant for health leaders, policy-makers, and scholars.

### Key Insights

• Continuing to perpetuate provider or profession-focused siloed models of care will not solve our HHR challenges—there will never be enough using past/current models.
• Not having our skilled workforce working at top of their scope and distributed equitably and aligned to population needs is no longer effective nor financially responsible.
• Using interprofessional or MDT needs-based frameworks will support the systemic paradigm shift needed to transition from a disease management bio-medical approach to a broader more holistic focus on health promotion and client-driven care.

For policy-makers building capacity in the system, key enablers were identified to effectively use any of these 9 evidence-informed MDT frameworks/models. They are considered as critical next steps.

### Critical enablers

#### Generate common current and longitudinal population needs-based health data

Generating current and longitudinal population health data will enable tracking health trends over time. Shifting care to a patient-driven, proactive, preventative approach from the current curative-reactive, bio-medical model will improve the health and wellness of the population over time and potentially shift traditional HHR workforce planning models, which typically focus on one or two professions, or a narrow or limited skill mix to a broader MDT.^1-4,13,14,17,18,22^ One framework encouraged planners to incorporate health promotion and preventative services to impact population health.^5^ The World Health Organization’s (WHO) *Workload indicators of staffing need manual* (2023) recognizes inefficiencies due to uncoordinated Human Resources for Health practices (HRH) and weak HRH coordination mechanisms, and weak human resources for health information systems.^14^

#### Create access to detailed human resource workforce supply data that includes availability, skills, and competencies

Access to consistent reliable data will inform a systematic approach to HHR workforce planning.^8,9,18,22,31,32^ Up-to-date databases of workforce supply are needed, listing not only individual staff types but also skills and competencies, the effective use of those skills, and their availability to work full-time equivalency. This data enriches HR systems to better distribute the skilled workforce according to population and regional needs.^15,16,24,25,27,28,31,33,34^ This data gap raises the profile and importance of interconnected technology and databases, not only for HR workforce planning and monitoring of population healthcare services, but also for workforce education and training.

Several of the frameworks’ authors discussed the need to distribute workforce capacity at a systems and population health level. That would develop more flexibility aligned to differences in needs and availability across population care settings and levels of care versus simply looking at the size of a population.^2,4,5,9,10,12,14-16,24-30^

Three of the four workforce planning models calculated a significant deficit of people to provide care when focused on care pathways limited to one subpopulation.^14,21,23,27^ One author discovered while using a competency-based, integrated needs-based framework across several professions that “there may be gaps in some professions, (but) there are enough people in the system to respond to a pandemic.”^12^ This reassurance by Murphy et al. will help leaders learn together to design and integrate MDTs differently to deliver people-centred health services.

#### Create a learning management system to support competency development and maintenance, performance support tools and resources driven by MDT skill mix

Some authors had concerns of an increasing gap between the patient care needs of the population and the competencies of the workforce.^6–8^ A systematic approach to determine models of care that match supply with demand by recruiting MDT members with the right skill mix to align the current and future workforce and the needs of the population^5,8-10,12,14,19,25,27,28,31,35^ would inform policy, HHR planning, and funding decisions for education and training. Some suggested competencies and learning needs change as people become more experienced and knowledgeable,^12,14,28^ highlighting the flexibility required when hiring, the adaptability needed among existing MDT members, and the resulting need to provide learning resources at the time of application or need.^26^ Authors also noted the need to integrate needs-based, service planning models that include both human and non-human resources.^6^ While we may not have considered technology as a team member, we now have an opportunity to expand our mental models of MDT integration and embrace technology as a formalized member of the team for support across the spectrum of care.

#### Align policies and governance to drive health system redesign to enable MDT learning, flexibility, and adaptability across the system

Policies and governance that drive health system redesign could help leaders build new MDT models with the flexibility and adaptability needed to respond to today’s health and HHR complexities. A phenomenon known as “map clinging dynamic” described by Black and Gregersen identifies “brain (mental) barriers” that impede strategic change when decision-makers keep the same approach that is not addressing current problems.^36^ As Bourgeault et al. states “Transformative change in healthcare delivery will forever remain challenging without aligning healthcare objectives, policy, and data.”^9^

### Strengths and limitations of this scoping review

This is the only published review in more than two decades that pulls together and summarizes MDT frameworks in one article and provides an analysis of the framework authors’ expert insights, learnings and “howling at the moon calls for action.” Given the commonalities in findings, health leaders and policy-makers benefit by applying the learnings as a template for (re-)designing their MDTs.

The limitations include searching for only English articles and a lack of common definitions for terms such as “healthcare team model of care frameworks” or “multidisciplinary team.” Other authors might have referred to these differently. Though there may be MDT frameworks/models published in other languages and in other cultures, the broad representation of professions, professionals, and population settings represented in the 9 frameworks resulted in such similar findings that health leaders can consider them generalizable and comparable.

## Conclusion

Having a summary of the published literature that describes population needs-based multidisciplinary frameworks gives health leaders and policy-makers options and a way forward to address the mismatch between need and service configuration. This scoping review offers a range of options and next-step considerations to inspire fresh and expanded thinking in making evidence-informed decisions about skill mix and optimal MDT composition.
